# At the bottom of the Pandora’s box: preserving AMR surveillance in Gaza’s collapse

**DOI:** 10.1128/msystems.01275-25

**Published:** 2026-01-30

**Authors:** Giancarlo Ceccarelli, Francesco Branda, Fabio Scarpa, Alberto Enrico Maraolo, Massimo Ciccozzi

**Affiliations:** 1 Department of Public Health and Infectious Diseases, University of Rome La Sapienza 119653, Rome, Italy; 2 Azienda Ospedaliero Universitaria Umberto I 18652 , Rome, Italy; 3 Genomics, AI, Bioinformatics, Infectious Diseases, Epidemiology Group (GABIE), Rome, Italy; 4 Migrant and Global Health Research Organization—Mi-Hero, Rome, Italy; 5 Medical Statistics and Molecular Epidemiology, University Campus Bio-Medico 9317, Rome, Italy; 6 Department of Biomedical Sciences, University of Sassari 390773, Sassari, Italy; 7 Section of Infectious Diseases, Department of Clinical Medicine and Surgery, University of Naples "Federico II" 60239, Naples, Italy; The University of Maine, Orono, Maine, USA

**Keywords:** policy, public health, global health, war context, conflict, Gaza Strip, MDRo, multidrug-resistant

## LETTER

Kumar et al. warn of severe antimicrobial resistance (AMR) in Palestine driven by prolonged conflict and health-system collapse, urging a ceasefire, restored humanitarian access, rebuilding water, sanitation, and hygiene/labs/vaccination, stronger stewardship/diagnostics, antibiotic regulation, and context-rooted policy (1).

In Gaza, however, the situation has deteriorated far beyond the conditions described just 2 months ago. Resources are reported to be exhausted, humanitarian access is obstructed, and the near-term outlook is bleak. In this context of complete resource depletion and no prospect of immediate aid, the otherwise valid priorities identified by the authors are, at present, unattainable, while the problem continues to escalate (2). More concerning still, beyond the direct clinical threat, is that system fragility has eliminated the capacity to measure the problem: with the loss of lab capacity, pharmaceutical supply chains, and operational tools, the data required to locate and quantify outbreaks have vanished. Without measurement, stewardship and outbreak control become conjectural, a de facto surrender to AMR and other communicable threats (3).

Yet even under such conditions, minimal functional capacity can and should be preserved. Ultralight syndromic surveillance—weekly aggregated counts of sentinel conditions (sepsis, infected wounds, acute respiratory infection, and diarrhea), combined with any available microbiological data, as well as deaths and critical stock-outs—can and should be collected at facility level and relayed to a central coordinating unit through available communication channels. Week-on-week trend analysis can provide an operational overview to guide the targeted allocation of scarce antibiotics, fluids, dressings, and infection-prevention resources.

Experiences from other protracted emergencies illustrate this potential. In Somalia, reactivating an electronic early warning and response network (EWARN) improved the timeliness of weekly reporting from approximately 25% to near universality over 4 years, with severalfold increases in alert verification (4). In Yemen, an electronic early-warning system became the *de facto* national communicable-disease backbone, covering around 2,000 facilities and sustaining weekly dashboards despite the collapse of routine surveillance (5). Regional analyses from the eastern Mediterranean show EWARN platforms meeting core timeliness thresholds and verifying a high proportion of alerts even under emergency conditions (6). In Syria, parallel early-warning systems functioned where routine systems had fractured, but coordination and data sharing proved decisive for outbreak control (7).

In Gaza, even with minimal operational capacity, the health system could identify priority needs through the systematic collection of standardized site-level data and their timely submission to a central coordinating body. Such aggregated information would enable the development of a shared operational overview, supporting the targeted allocation of scarce resources (Table 1). While lacking diagnostic precision, this approach restores situational awareness, an essential foundation for any AMR response. Measurement is not a luxury; it is the essential first act in regaining control during an escalating health emergency.

**TABLE 1:** Minimal, lab-independent epidemiologic package for AMR-relevant surveillance in conflict settings^*a*^

Information type	Minimal variables (operational definitions)	Source and frequency	Quality standards (minimum thresholds)	Analysis and alert thresholds	Immediate actions (IPC/AMS)
Priority clinical syndromes	Weekly case counts by ward for severe sepsis/shock, infected wounds/SSI, ARI/pneumonia, and acute watery diarrhea (stratify <5 years/≥5 years)	Ward registers → weekly	Timeliness ≥80% sites on time, completeness ≥95% required fields	Compare to 8-week median, trigger alert if >2 SD above baseline or week-on-week doubling	Rapid clinical audit, reinforcement of hand hygiene, checking theater/sterilization pathways
Outcomes	Inward deaths (sepsis/trauma-infected), antibiotic switch ≤72 h (empiric failure), 7-day readmissions for infection	Clinical log → weekly	Internal coherence (deaths ≤ admissions), ≥95% completeness	Joint rise in deaths + early switches ⇒ operational alert, expressed as rates per 100 admissions	Review of the empiric regimen, escalation/de-escalation per local guidance
Risk factor mix	Prior antibiotics (30 days: yes/no); devices (line, catheter); ICU/OR in the past 7 days; conflict-related wound	Triage sheet → weekly aggregate	≥90% completeness	Cluster-specific increases (e.g., SSI among external-fixator patients) ⇒ targeted alert	Cohorting, device-bundle checks, bedside education
Operational denominators	Admissions, bed-days by ward; number of surgeries	Admin summary → weekly	Plausible series (no “heaping”)	Compute rates/1,000 bed-days for site/ward comparisons.	Resource reallocation, surge support
Antibiotic use (PPS)	For each patient on antibiotics: diagnosis, regimen, route, AWaRe class, indication (empiric/targeted), intended duration	Quarterly PPS (1–3 days)	Standard PPS forms, double-check 10% entries	Watch/reserve >50% or surgical prophylaxis >24 h ⇒ AMS alert	De-escalation, IV-to-oral switch, stopping prophylaxis >24 h
Pharmacy consumption (AMC)	DDD or units per 100 bed-days by molecule, stock-outs (days without stock)	Pharmacy → monthly	Series continuity ≥90%, reconcile with bed-days	Rising watch/reserve share or prolonged stock-outs ⇒ protocol review	Adjustment of empiric protocols, prioritized resupply
Event-Based Surveillance (EBS)	Rumor log of unusual events (who/what/where/when), ≥2 similar signals/72 h in same ward/area	Continuous, weekly synthesis	Traceable verification for ≥80% signals	Threshold met ⇒ rapid field check within 24–48 h	Bedside verification, immediate IPC measures if confirmed
System status	Days open; staffing on duty; power outages >4 h; WASH/PPE disruptions	Weekly	Stability: <10% weeks missing reports	Outlier downtime ⇒ red status in dashboard	Targeted support, redistribution of workload
Data quality and evaluation	Timeliness, completeness, duplicates, inconsistencies, PPV of alerts (verified/issued)	Coordination hub → weekly review	Targets: timeliness ≥80%, completeness ≥95%, duplicates <10%; PPV trending upward month-to-month	Flag sites below threshold, microtraining	Feedback to sites, 15-min microaudit

^*a*^ This minimalist epidemiologic package is derived from WHO EWAR/EWARS operational guidance and CDC/ECDC surveillance-evaluation frameworks and is informed by peer-reviewed evaluations of EWAR/IDSR performance in conflict settings (Yemen, Somalia, and South Sudan), with stewardship modules aligned to Global-PPS and WHO GLASS-AMC methodologies. Abbreviations: AMC, antimicrobial consumption; AMS, antimicrobial stewardship; ARI, acute respiratory infection; AWaRe, Access/Watch/Reserve antibiotic classification; CDC, US Centers for Disease Control and Prevention; EBS, event-based surveillance; ECDC, European Center for Disease Prevention and Control; EWAR, Early Warning Alert and Response; EWARS, Early Warning Alert and Response System; GLASS, Global Antimicrobial Resistance and Use Surveillance System; IDSR, Integrated Disease Surveillance and Response; IPC, infection prevention and control; PPS, point prevalence survey; PPV, positive predictive value; SSI, surgical site infection; WHO, World Health Organization. See references 8–13.

Critically, the international community must recognize that microbes are indifferent to borders: without coordinated surveillance, safe medical corridors, and harmonized infection-prevention measures, resistant organisms now entrenched in Gaza will inevitably spread regionally and beyond. Addressing Gaza’s AMR crisis is a matter of public health, not politics.
